# World Social Health Insurance: Strengthening Health Systems in Low-Income Countries

**DOI:** 10.1371/journal.pmed.0040137

**Published:** 2007-03-27

**Authors:** Wim Van Damme

The paper by Ooms et al. [1] is very timely and stimulating. It launches the debate on how to finance globally the right to health care in low-income countries. This is a most welcome step, going beyond the usual aspirational rhetoric. I would like to contribute to the development of this idea.

First: the name. I fear that “World Health Insurance” may create confusion. Health insurance can be a pure risk-sharing mechanism without built-in solidarity between rich and poor, healthy and less healthy, or between old and young. But the concept of social health insurance—as the statutory health insurance systems in much of continental Europe are usually referred to—is intrinsically based on such solidarity, which certainly is one of the values underpinning Ooms' proposal. I therefore propose the name “World Social Health Insurance.”

Second: the contribution by low-income countries. Ooms et al. propose 15% of government budget as a fair contribution, the so-called Abuja target. I fear, however, that this target does not create the right incentive for governments in low-income countries, many of whom are reluctant or unable to tax their citizens, even the richer ones, and fail to create a decent tax basis. Consequently, some governments have extremely lean budgets, even below 20% of gross domestic product (GDP) [2], while the World Bank estimates that at least some 30% of GDP is needed to sustain a well-functioning state. I therefore think that calculating the contribution of low-income countries to their countries' health system as 4% or 5% of GDP would constitute a fairer burden sharing mechanism.

Third: the contribution of high-income countries. Ooms et al. propose that rich countries adopt a burden sharing similar to their contribution to World Bank's IDA 14 (the 14th replenishment of the International Development Association). This normalizes the low commitment of donors such as the United States, contributing in absolute terms hardly more than the United Kingdom or Japan, while its total GDP is much larger. I therefore think that for high-income countries, a contribution linked to total GDP would be fairer: e.g., 0.15%, which would be a bit more than one-fifth of the 0.7% target that most OECD countries have committed to as total overseas development assistance. Alternatively, and more in line with the concept of social health insurance, high-income countries could dedicate a share of domestic health expenditure (e.g., 1%) to world social health insurance. With total health expenditure in the United States now reaching US$2,000 billion [3], this modest 1% would already come close to the total needs as estimated by Ooms.

Lastly: operationalization. How to operationalize the massive scale-up of services proposed, given present human resource constraints and institutional capacities, is still a huge challenge. Whether it is best to take inspiration from the experience with rounds of competitive proposals, followed by performance-related disbursement, as the Global Fund uses, or whether the proposal of the Global Alliance for Vaccines and Immunisation (GAVI) to link disbursement to strategic government plans and sector-wide approaches would be more successful, remains to be explored.

We sincerely hope that the idea launched by Ooms et al. catches on, so that health services in low-income countries can rapidly expand. This can be seen, as Garrett convincingly argues [4], as an expression of a moral duty, as a form of public diplomacy, or as an investment in self-protection. Whatever the drive, there are enough reasons to start preparing it backed by long-term reliable funding, fairly shared between all stakeholders, according to their purchasing power.
